# Comparative evaluation of nano-hydroxyapatite dentifrice, diode laser alone and combined for dentin hypersensitivity: A randomized clinical trial

**DOI:** 10.6026/973206300222380

**Published:** 2026-04-30

**Authors:** Ekata Jaindesarda, Lisa Chacko, Ruhi Subhash Sankpal, Farhan Abdul Azeez, Sanyukta Singh, Maithili Madhukar Kshirsagar

**Affiliations:** 1 Private Practitioner, Periodontists, Ace dental care, Pune, India; 2 Department of Periodontics, SMBT Dental College & Hospital, Sangamner, Maharashtra, India; 3 Department of Conservative Dentistry and Endodontics, Bharati Vidyapeeth (Deemed to be University) Dental College and Hospital, Sangli, Maharashtra; 4 Registrar Endodontics, Al Hammadi Hospital, Prince Muqrin Ibn Abdulaziz St, An Nuzhah, Riyadh, Kingdom of Saudi Arabia; 5 Department of Conservative Dentistry and Endodontics, Saveetha Dental College and Hospitals, SIMATS, Chennai, Tamil Nadu, India; 6 Department of Oral Medicine & Radiologist, Nanded Rural Dental College and Research Center, Nanded, India

**Keywords:** Dentin hypersensitivity (DH), desensitizing agents, diode laser, nano-hydroxyapatite (N-HAP)

## Abstract

Dentin hypersensitivity is a common clinical condition characterized by short, sharp pain arising from exposed dentinal tubules, posing a challenge for long-term management. This randomized, single-blind parallel-arm clinical trial evaluated and compared the effectiveness of nano-hydroxyapatite (N-HAP) dentifrice and diode laser used alone and in combination, with fluoride dentifrice as a control. Fifty-five hypersensitive sites were allocated into four groups and sensitivity was assessed using VAS and VDS scores at baseline, immediately post- treatment and at 1, 4 and 8 weeks. All test groups demonstrated a significant reduction in dentin hypersensitivity compared to the control group, with the combination therapy showing the greatest and most sustained reduction. The combined use of N-HAP dentifrice and diode laser exhibits a superior synergistic effect, indicating its potential as an effective approach for managing dentin hypersensitivity.

## Background:

Dentin hypersensitivity (DH) is one of the most common complaints reported by dental patients, who greatly influences the quality of life of patients & defined as short and intense pain occurs in correspondence with the exposed dentine as a response to thermal, chemical, tactile or osmotic stimuli and cannot be attributed to any other tooth defect or pathology [1, 2]. The hydrodynamic theory remains the most widely accepted explanation, attributing pain to fluid movement within open tubules [3]. Traditional desensitizing agents such as potassium nitrate and sodium fluoride aim to occlude tubules or desensitize nerves but often show limited or temporary effectiveness [4]. Conventional desensitizing agents such as potassium nitrate and sodium fluoride act by occluding tubules or desensitizing pulpal nerves. However, their effects are often limited or short-lived [4]. Among newer materials, nano- hydroxyapatite (N-HAP) has demonstrated superior adherence to enamel compared to conventional hydroxyapatite [5]. The formation of a nano-layer on the enamel surface not only protects against mineral loss but also provides acid resistance due to its insolubility and bioactive nature [6]. Owing to its smaller particle size, high surface area, excellent biocompatibility and remineralization potential, N-HAP has been widely used in toothpaste formulations. Furthermore, its anti- cariogenic properties and ability to occlude dentinal tubules make it a promising candidate for managing DH [7, 8]. The effects of desensitizing agents are not definitive and when added to daily tooth brushing and interference from acidic foods, these agents may easily lose their power to obliterate the dentinal tubule, so that their effectiveness diminishes with time [9, 10]. Therefore, the literature shows that no desensitizing agent or forms of treatment have been effective in all patients or had a long- lasting effect [11]. Low-level diode lasers (especially 940 nm) have emerged as viable alternatives, capable of inducing tertiary dentin formation, photobiomodulation and protein coagulation [12]. Despite their effectiveness, evidence on their long-term performance remains inconsistent which ranging from 5.2% to 100% [13]. Recently, it has also been used in combination with desensitizing agents such as strontium fluoride, sodium fluoride [14, 15], stannous fluoride [16], Nova Min [17], potassium oxalate [18], dentin bonding agent [19], potassium nitrate [16] etc., to test their combined efficacy in the management of DH [9, 20, 21, 23- 24]. The better performance of DH is primarily because of higher adhesion to tubule in presence of lasers energy as it induces melting of superficial layers leading to longer tubule occlusion and reduction in DH related pain as reported by Siddiqui *et al.* [25]. The clinical effect of combined application of N-HAP and diode laser in the management of DH has not been explored adequately. Therefore, it is of interest to bridge this gap by evaluating the efficacy of the N-HAP dentifrice & diode laser used alone or in combination when compared to positive control in the management of DH.

## Methodology:

A randomized, parallel-arm, single-blind clinical trial was conducted at the Department of Periodontology and Oral Implantology. Sample size was calculated using Open Epi 3.01 software, maintaining 80% power and 95% confidence interval.

A total of 55 hypersensitive sites were identified and randomly allocated to four groups:

[1] Group I: N-HAP dentifrice

[2] Group II: 940 nm diode laser

[3] Group III: N-HAP dentifrice + diode laser

[4] Group IV: Fluoride dentifrice (control)

Inclusion criteria included patients aged 19-60 years with clinically diagnosed DH, good oral hygiene, non-carious cervical lesions <2 mm and VAS baseline scores > 4 in the facial aspects of the incisors, canines and premolars. Exclusion criteria included systemic diseases, active caries and recent periodontal or desensitizing treatments in the last 3 months, pregnancy and enamel defects. Treatment was administered once weekly for 3 weeks. The diode laser was applied in non-contact mode (0.2 W, 940 nm, 30s x 2 per site with a 10s interval); while the pea-sized desensitizing agent was taken on the applicator tip dentifrices were applied on the surface of the tooth for 15 minutes. Post operatively participants were instructed to avoid rinsing, eating or drinking for 30 minutes post-application and refrain from mechanical plaque control for 12 hours. Participants were instructed to brush twice daily using a soft-bristled toothbrush and non-desensitizing toothpaste with the Modified Bass technique. They were also advised to maintain proper oral hygiene, avoid acidic foods and brush before meals when dietary acids were identified as the primary cause of DH. Clinical evaluations using VAS and VDS were recorded at baseline, immediately post-treatment and at 1, 4 and 8 weeks. Statistical analysis was performed using ANOVA and post hoc tests, with significance set at p<0.05.

## Results:

Thirty-two participants (mean age: 38.75 years; 15 males, 17 females) were equally assigned to four groups: N-HAP, diode laser, combination and control. At baseline, no significant intergroup differences were observed (p > 0.05). Gender distribution was balanced; central incisors were the most affected teeth overall (41.8%) (Table 1 - see PDF). All groups except control showed significant reductions over time (p < 0.001), with Group II (diode laser) exhibiting the greatest decline by 4 weeks (0.23 ± 0.38) (Table 2 - see PDF). Significant reduction in all groups (p < 0.001); Group III (combination) showed the highest reduction from 6.25 ± 1.18 to 0.60 ± 0.15 (90%), followed by Group II (90%), Group I (59%) and Group IV (41%) (Table 3 - see PDF) (Figure 1 - see PDF). Group III again showed maximum improvement (6.92 ± 1.03 to 1.01 ± 0.36; 85%), followed by Group II (73.7%), Group I (65%) and Group IV (47.6%) (Table 3 - see PDF) (Figure 2 - see PDF). All groups improved (p < 0.001); Group II showed the greatest reduction (6.0 to 0.15; 97%), followed by Group III (88.4%), Group IV (65.5%) and Group I (59%) (Table 4 - see PDF) (Figure 3 - see PDF). Intergroup comparisons confirmed Group III's superiority (p < 0.001**). Where Group III led (7.28 to 1.21; 83.3%), followed by Group II (72%), Group I (67%) and Group IV (41.6%) (Table 4 - see PDF) (Figure 4 - see PDF). Overall, the combination therapy of N-HAP and diode laser was most effective in reducing DH, followed by diode laser alone, N-HAP dentifrice and control.

## Discussion:

The study findings demonstrate that all treatment modalities significantly reduced DH compared to the control, consistent with previous studies. The combination of N-HAP and diode laser achieved the greatest reduction, validating the synergistic hypothesis. Despite decades of research, dentinal hypersensitivity (DH) treatment remains largely empirical, with most therapies failing to meet the ideal criteria outlined by Grossman (1935) [26]. Adhering to the trial design recommendations of Holland *et al.* (1997) [2], this randomized, parallel, single-blind study compared the efficacy of N-HAP dentifrice, diode laser therapy and their combination against a fluoride-based control, which served as a positive benchmark despite not being a desensitizing agent [27, 28]. N-HAP's mechanism involves tubule occlusion via hydroxyapatite deposition and remineralization [5, 6]. Diode laser therapy enhances desensitization through photobiomodulation, nerve depolarization blockade and increased tertiary dentin formation [1, 29]. When combined, the laser likely facilitates deeper penetration and adhesion of N-HAP particles, improving long-term effectiveness [30, 31]. The study included participants aged 19-60 years (mean 38.75), with DH more prevalent in the third and fourth decades, likely due to oral hygiene and diet. Females slightly outnumbered males (53.13% vs. 46.87%), consistent with some studies suggesting higher DH prevalence in females, possibly from greater oral care and dental visits. DH most commonly affected central incisors (41.8%), canines (29%) and first premolars (16.4%), mainly on buccal surfaces, aligning with prior reports. Stimuli used to assess DH were initially associated with fluid movement within dentinal tubules [32]. However, not all stimuli reliably quantify DH; tactile, cold and evaporative air stimuli are recommended due to their physiological relevance and greater experimental control [33, 34]. Holland *et al.* [2] advised using multiple stimuli, starting with the least severe and allowing sufficient intervals to avoid interaction effects. This study used tactile followed by air blast stimuli, with a 3-minute gap to minimize cross-stimulus influence, given the unknown pulpal recovery time. DH was assessed at baseline, immediately post-treatment and at 1, 4 and 8 weeks using Visual Analog Scale (VAS) and Verbal Descriptor Scale (VDS) in response to both stimuli. Defect-specific plaque index showed significant improvement from baseline 1, 4 and 8 weeks, reflecting better oral hygiene, assessed using the Silness and Loe Plaque Index [35]. Plaque accumulation leads to acid production causing tooth demineralization and dentinal tubule exposure, contributing to dentinal hypersensitivity (DH) [36, 37-38]. All groups showed reduced plaque scores post-treatment, likely due to regular maintenance and patient motivation. Good plaque control is generally linked to reduce DH [39, 40], through some studies report no clear correlation [41], indicating the relationship remains controversial. Dentine hypersensitivity (DH) was assessed using both the Visual Analog Scale (VAS) and Verbal Descriptor Scale (VDS), which measure patients' perceived pain intensity. VAS, a 0-10 cm line, is widely validated for its sensitivity and reliability in capturing subtle changes in pain, making it ideal for DH evaluation [42, 43, 44-45]. VDS uses descriptive terms to quantify pain and is reliable across age groups, especially when assessing chronic conditions [44, 46 and 47]. In this study, both scales correlated well across stimuli, supporting their combined use for consistent and effective pain assessment in DH. For tactile hypersensitivity tactile stimuli, applied using a sharp explorer in a mesio- distal direction, help identify DH by triggering pain via fluid shifts in dentinal tubules. Through less invasive than thermal methods, it can still provoke a response in sensitive teeth and is a standard clinical assessment tool [48, 49, 50, 51, 52-53]. In cases of air blast hypersensitivity air blast combines thermal and evaporative effects, inducing rapid outward fluid shifts and triggering pain via mechanoreceptor activation [53, 54]. In this study, a 1-second air blast from a three- way syringe was used to avoid overstimulation while effectively eliciting DH-related pain. In the present study, Group III-treated with a combination of nano-hydroxyapatite (N-HAP) dentifrice and diode laser (DL) - demonstrated the most significant reduction in dentinal hypersensitivity (DH), with 90% reduction in VAS tactile, 85% in VAS air blast, 88.4% in VDS tactile and 83.3% in VDS airblast scores after 8 weeks. This synergistic efficacy may be attributed to both the occlusive effect of N-HAP and the neural modulation by DL. N-HAP enhances remineralization through its biomimetic structure and physicochemical properties, forming a protective enamel-like layer (~1-2 µm thick) that occludes dentinal tubules [5, 29]. Studies by Wang *et al.* (2024) [6] and Rathi *et al.* [55] confirmed N-HAP's superior remineralization under acidic conditions. DL, particularly at 940 nm, reduces DH through mechanisms such as neural depolarization inhibition and photobiomodulation [1, 29]. The 1-week VAS score reduction in Group III (80.6%) aligns with findings by Siddiqui *et al.* (2022) [25], who reported a 77.9% reduction at 1 week and 90% at 2 weeks using the same combination. Support *in vitro* studies Neel *et al.* [30], Pushkar *et al.* (2025) [56], Shamel and Banna (2022) [57] highlight durable tubular occlusion and acid resistance with this dual approach. Group II (DL alone) also showed substantial improvement: 90% (VAS tactile), 73.7% (VAS airblast), 97% (VDS tactile) and 72% (VDS airblast) at 8 weeks. These outcomes mirror prior research by Hugar *et al.* (2023) [58] and are consistent with studies by Pourshahidi *et al.* (2019) [59], Raichur *et al.* (2013) [21] and Tocarruncho *et al.* (2018) [60], all demonstrating DL's efficacy over 4 weeks. Further, *in vitro* results from Rodriguez (2024) [61] and Umana *et al.* (2013) [62] validate DL's tubule-sealing capabilities. Group I (N-HAP alone) recorded moderate reductions (59% VAS tactile, 67% VAS airblast), consistent with Vano *et al.* & Vano *et al.* (2014, 2018) [63, 64] and Gopinath *et al.* (2015) [65]. SEM analyses from Kulal *et al.* (2016) [66] confirmed high tubule occlusion (up to 98.1%). Group IV (fluoride control) showed the least improvement, with 41-47.6% VAS reductions. While Holland *et al.* (1997) [2] recognized fluoride as a benchmark, evidence from Sharma *et al.* (2013) [67] and Vano *et al.* (2018) [64] suggests low efficacy at 0.15% fluoride ion. To summarise, the N-HAP + DL combination emerged as the most effective modality for rapid and sustained DH relief, outperforming monotherapies and offering a promising non-invasive treatment strategy. This study provides clinical evidence supporting the synergistic effect of nano-hydroxyapatite dentifrice combined with a 940-nm diode laser in the management of dentin hypersensitivity. The combined therapy demonstrated greater and more sustained reduction in hypersensitivity compared to either modality used alone or fluoride dentifrice. The findings contribute to existing literature by establishing a non-invasive, clinically effective combination approach for short-term management of dentin hypersensitivity. This study has several limitations affecting result interpretation and generalizability. The small sample size and 20% dropout rate reduced statistical power and internal validity. The lack of a placebo control limits differentiation of true treatment effects from placebo responses. The short duration prevents assessment of long-term outcomes. The single-blind design may introduce observer bias and reliance on subjective measures (VAS, VDS) could affect data consistency. Future research should involve larger samples, longer follow-up, double-blind designs, objective measures and improved retention to validate these findings.

## Conclusion:

All test groups showed significant reductions in dentin hypersensitivity scores, with diode laser therapy outperforming nano- hydroxyapatite alone and the combination therapy producing the greatest and most consistent improvement. Although defect-specific plaque index decreased in all groups, hypersensitivity reduction was significantly greater in the test groups compared to the control. The combined use of nano-hydroxyapatite dentifrice and diode laser provided the most effective and sustained relief over 8 weeks. However, this should be interpreted in light of the short follow-up period and limited sample size.

## References

[1] Bekes K, Hirsch C (2013). Clin Oral Investig..

[2] Holland GR (1997). J Clin Periodontol..

[3] Brännström M (1967). Caries Res..

[4] Dilsiz A (2010). Photomed Laser Surg..

[5] Li L (2008). Cryst Eng Comm..

[6] Wang Y (2024). iScience..

[7] Huang S (2010). J Dent..

[8] Huang S (2011). Caries Res..

[9] Kerns DG (1991). J Periodontol..

[10] Panduric V (2001). J Oral Rehabil..

[11] Lopes AO, Aranha AC (2013). Photomed Laser Surg..

[12] Machado AC (2018). Lasers Med Sci..

[13] Kimura Y (2000). J Clin Periodontol..

[14] Lan WH (1999). J Endod..

[15] Kumar NG, Mehta DS (2005). J Periodontol..

[16] Hines D (2019). J Am Dent Assoc..

[17] Bm S (2014). J Clin Diagn Res..

[18] Vieira AH (2009). Photomed Laser Surg..

[19] Tengrungsun T, Sangkla W (2008). J Dent..

[20] Ranjan R (2013). J Dent Lasers..

[21] Raichur PS (2013). Gen Dent..

[22] Gojkov-Vukelic M (2016). Med Arch..

[23] El Mobadder M (2019). Dent J (Basel]..

[24] Liu Y (2013). J Lasers Med Sci..

[25] Siddiqui S (2022). J Conserv Dent..

[26] Grossman L (1935). J Am Dent Assoc..

[27] Pearce NX (1994). J Periodontol..

[28] Gómez VC (2026). J Dent..

[29] Cotler HB (2015). MOJ Orthop Rheumatol..

[30] Abou Neel EA (2024). Appl Phys A..

[31] Siddiqui S (2024). J Pharm Bioallied Sci..

[32] West NX (2013). Clin Oral Investig..

[33] Orchardson R, Gillam DG (2006). J Am Dent Assoc..

[34] Gillam DG, Newman HN (1993). J Clin Periodontol..

[35] Löe H (1967). J Periodontol..

[36] Wolff MS (2009). Adv Dent Res..

[37] Trowbridge HO, Silver DR (1990). Dent Clin North Am..

[38] Cox CF. (1994). Am J Dent..

[39] Haneet RK, Vandana LK (2016). Int Dent J..

[40] Kawasaki A (2001). J Oral Rehabil..

[41] Taani DQ, Awartani F (2001). Quintessence Int..

[42] Martínez-Ricarte J (2008). Med Oral Patol Oral Cir Bucal..

[43] Lund RG (2013). Acta Odontol Scand..

[44] Freitas BLS (2020). Cad Saude Colet..

[45] Gagliese L (2005). Pain..

[46] Gaston-Johansson F (1990). J Pain Symptom Manage..

[47] Ware LJ (2006). Pain Manag Nurs..

[48] Biagi R (2016). Ann Stomatol (Roma)..

[49] Porto ICCM (2009). J Oral Sci..

[50] Öncü E (2017). Microsc Res Tech..

[51] Karim BF, Gillam DG (2013). Int J Dent..

[52] Camps J, Pashley D (2003). J Periodontol..

[53] Pashley DH (1996). Arch Oral Biol..

[54] Matthews WG (1993). Arch Oral Biol..

[55] Rathi N (2017). J Clin Exp Dent..

[56] Pushkar A (2025). Cureus..

[57] Shamel M, Banna SE (2022). J Stomatol..

[58] Hugar S (2023). Int J Community Med Public Health..

[59] Pourshahidi S (2019). Clin Oral Investig..

[60] Tocarruncho O (2018). Univ Odontol..

[61] Rodriguez CGB (2025). Lasers Med Sci..

[62] Umana M (2013). Photomed Laser Surg..

[63] Vano M (2014). Quintessence Int..

[64] Vano M (2018). Clin Oral Investig..

[65] Gopinath NM (2015). J Int Oral Health..

[66] Kulal R (2016). J Clin Diagn Res..

[67] Sharma D (2013). J Clin Dent..

